# Short-Term Efficacy and Safety of Secukinumab for Ankylosing Spondylitis: A Systematic Review and Meta-Analysis of RCTs

**DOI:** 10.1155/2020/1639016

**Published:** 2020-10-26

**Authors:** Yu Zhou, Jinhui Ma, Juncheng Ge, Bailiang Wang, Debo Yue, Weiguo Wang

**Affiliations:** ^1^Department of Orthopaedic Surgery, Peking University China-Japan Friendship School of Clinical Medicine, Beijing 100029, China; ^2^Department of Orthopaedic Surgery, Center for Osteonecrosis and Joint Preserving & Reconstruction, China-Japan Friendship Hospital, Beijing 100029, China

## Abstract

Secukinumab is a novel IL-17A inhibitor that has been confirmed to be effective for treating PsA and RA. Several studies have demonstrated that secukinumab also provides benefits for AS patients. Thus, we performed a meta-analysis of RCTs to evaluate the short-term efficacy and safety of secukinumab for the management of AS. The PubMed, Medline, Embase, Web of Science, and Cochrane Library databases were searched for RCTs published prior to March 2020 on the treatment of AS with secukinumab. The primary outcome was the ASAS20 response, and the secondary outcomes included the ASAS40 response, ASAS5/6 response, SF-36 PCS score, ASQoL score, and AEs. Dichotomous data were expressed as pooled RRs with 95% CIs, while continuous data were expressed as pooled MDs with 95% CIs. Subgroup analysis was conducted based on whether the AS patients previously underwent treatment with TNFi. A total of 4 RCTs with 1166 patients were included in our meta-analysis. At week 16, secukinumab 150 mg yielded significant improvements in the clinical response and patient-reported outcomes for AS patients. There was no increased risk of AEs. Consistent results were detected in the meta-analysis of secukinumab 75 mg versus a placebo. Furthermore, no significant difference was detected between the secukinumab 75 mg group and secukinumab 150 mg group. We concluded that secukinumab is effective for treating AS and generally well tolerated by AS patients in the short term, regardless of whether they previously underwent TNFi treatment. The superiority of secukinumab 150 mg over secukinumab 75 mg seems to be limited, since no significant difference in any endpoint was detected between the two groups.

## 1. Introduction

Ankylosing spondylitis (AS) is a chronic autoimmune inflammatory disease and a subtype of radiographic axial spondyloarthritis [1]. The prevalence of AS in the Chinese population is 0.29% and continues to increase; the ratio of males to females who are affected by this disease is 2.8 : 1 [2]. AS predominantly affects the spine and sacroiliac joints, causing chronic back pain, fatigue, and a loss of spinal mobility [3]. Without effective treatment, due to excessive bone regeneration and syndesmophyte formation, the gaps between joints gradually become filled, ultimately leading to ankylosis, deformities, a poor quality of life, and a substantial economic burden on patients and society [4, 5].

To date, the underlying mechanism of AS has not been identified completely. In recent years, published studies have uncovered several potential factors contributing to the occurrence of AS, such as genetic features, intestinal microbiomes, infections, as well as medication, and toxin exposure [6]. The HLA-B27 allele represents the strongest genetic factor, and there are four mainstream hypotheses attempting to illustrate its role in the pathogenesis of AS, including the IL-23/IL-17 axis theory [7]. Previous studies have demonstrated that AS patients exhibit increased IL-17 and IL-23 levels in the serum and synovial fluid [8, 9]. This imbalance of the IL-17/IL-23 axis may lead to uncontrolled inflammation, joint destruction, and excessive bone formation, which can promote the progression of the disease [4, 10, 11]. In addition, the blockage of IL-17 has been proved to reduce cartilage destruction and bone erosion in collagen-induced arthritis models, which suggests that IL-17 should potentially be a target for the management of AS [12, 13]. Taken together, these results suggest that AS is an autoimmune disease caused by the interaction between host genetic features and environmental factors, and the IL-23/IL-17 axis plays a critical role in its pathogenesis.

Historically, the treatment of AS has been limited to nonsteroidal anti-inflammatory drugs (NSAIDs). The introduction of tumor necrosis factor inhibitors (TNFis) has opened a new chapter and driven significant advancements in the management of AS over the past 20 years [14]. However, TNFis have their own limitations since 40% of AS patients have failed to respond adequately [15]. Secukinumab is a novel IL-17A inhibitor that has been confirmed to be effective for treating psoriatic arthritis (PsA) and rheumatoid arthritis (RA) [16, 17]. Several randomized controlled trials (RCTs) and studies conducted in real-world settings have demonstrated that secukinumab also provides benefits for patients with AS [18–25]. However, its clinical application is limited because current evidence is not as robust as that for other therapies (NSAIDs and TNFis). Therefore, we conducted a meta-analysis of RCTs to evaluate the short-term efficacy and safety of secukinumab for treating AS.

## 2. Materials and Methods

This meta-analysis was performed in accordance with the Preferred Reporting Items for Systematic Reviews and Meta-Analyses (PRISMA) reporting guidelines [26] for conducting meta-analyses of intervention trials.

### 2.1. Search Strategy

We searched PubMed, Medline, Embase, Web of Science, and Cochrane Library for available articles published prior to March 2020 on the treatment of AS with secukinumab. The search terms were as follows: “ankylosing spondylitis”, “spondyloarthritis”, “secukinumab”, and “cosentyx”. No publication status restrictions were applied. For this meta-analysis, we considered only studies published in English. Furthermore, we also searched the reference lists of review articles for potentially relevant trials.

### 2.2. Inclusion Criteria

The inclusion criteria were as follows: the patients were diagnosed with AS on the basis of the 1984 modified New York diagnostic criteria; at least one experimental group was treated with secukinumab, while the control group was treated with a placebo; the efficacy of secukinumab for the treatment of AS was assessed; the primary outcome was the proportion of patients who met the ASAS20 criteria at week 16, and the secondary outcomes included the ASAS40 response rate, ASAS5/6 response rate, the change from baseline in the scores of the Short Form-36 Physical Component Summary (SF-36 PCS), and Ankylosing Spondylitis Quality of Life Scale (ASQoL) at week 16. Studies were included if at least one outcome was reported. Only studies that were designed as randomized controlled trials were included. Articles of other publication types were excluded.

### 2.3. Study Selection and Data Extraction

Two authors screened all the titles and abstracts independently to identify the studies that met the inclusion criteria. Then, the full texts of all the potentially relevant articles were reviewed to assess their eligibility. When multiple articles describing the same trial were published, the most complete article was included, and disagreements were settled through discussion.

Two authors extracted data using a standard form, and a third reviewer verified its accuracy. The following data were extracted from the eligible articles: first author and publication year, study design, characteristics of patients (sex, weight, age, and duration of AS), intervention, and outcomes. Data reported in other formats (mean ± SE) were transformed into the mean ± SD format in accordance with the Cochrane Handbook recommendations.

### 2.4. Quality Assessment

Two authors independently evaluated the methodological quality of the included studies in accordance with the Cochrane quality assessment tool based on seven items: random sequence generation, allocation concealment, blinding of the participants and personnel, blinding of the outcome assessment, incomplete outcome data, selection reporting, and other bias. The studies were considered to have a “low risk of bias”, an “unclear risk of bias”, or a “high risk of bias” based on the evaluation criteria, and disagreements were settled by a third author.

The quality of evidence was assessed by two authors independently in accordance with the Grading of Recommendations Assessment, Development, and Evaluation (GRADE) criteria [27] based on five items: risk of bias, inconsistency, indirectness, imprecision, and publication bias. The level of evidence was graded based on four grades: “high quality”, “moderate quality”, “low quality”, or “very low quality”. High quality indicated that additional research is very unlikely to change our confidence in the estimate of the effect, moderate quality indicated that additional research is likely to have an important impact on our confidence in the estimate of the effect and may change the estimate, low quality indicated that additional research is very likely to have an important impact on our confidence in the estimate of the effect and is likely to change the estimate, and very low quality indicated that we are very uncertain about the estimate.

### 2.5. Statistical Analysis

All statistical analyses were performed using Review Manager (RevMan 5.3, Cochrane Collaboration, Copenhagen, Denmark). Dichotomous data were expressed as risk ratios (RRs) with 95% CIs, and continuous data were expressed as mean differences (MDs) with 95% CIs. The heterogeneity of the included studies was evaluated by the *I*^2^ index. Random-effects models were used in all of the meta-analyses. Statistical significance was defined as *P* < 0.05. Subgroup analysis was conducted based on whether the patients previously underwent TNFi treatment. Sensitivity analysis was conducted by removing one single study at a time to explore the impact of an individual study.

## 3. Results

### 3.1. Study Selection

**Figure 1:**
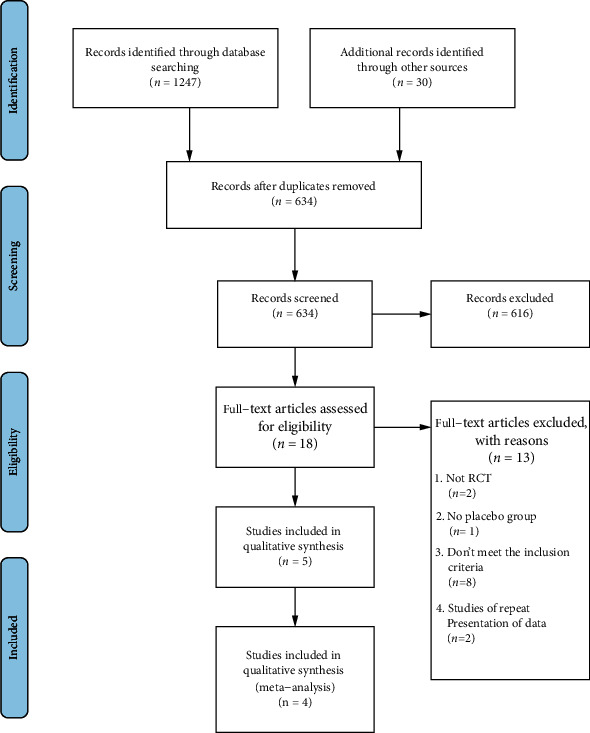
Flow chart of the literature search.

The steps of study selection were summarized in Figure 1. After the initial search, a total of 1247 articles were identified, 613 of which were ruled out due to duplication. A total of 616 studies were excluded after the titles and abstracts were read. Then, the full texts of the remaining 18 articles were retrieved for further assessment, and 13 articles were eliminated for the following reasons: the study was not an RCT, there was no placebo group, the study did not meet the inclusion criteria for outcomes, or it was a duplicate of a previous publication. Among the 5 studies [18–22] that met our inclusion criteria, one [18] was excluded because it used a different method of administration of secukinumab. Ultimately, 4 RCTs were included in the quantitative analysis [19–22].

### 3.2. Characteristics of the Included Studies

The characteristics of the included trials and patients were shown in Table 1. All of the studies were randomized, double-blind, placebo-controlled, multicenter phase 3 trials. The patients with active AS met the modified New York criteria and were over 18 years old. The other inclusion criteria included a score of at least 4 points on the bath ankylosing spondylitis disease activity index (BASDAI) and a score of at least 4 cm on a 10 cm visual analogue scale (VAS) for spinal pain, regardless of whether the patients were being treated with the maximum tolerated doses of NSAIDs. A history of treatment with disease-modifying and antirheumatic drugs (DMARDs) was permitted. The patients could continue to receive sulfasalazine, methotrexate, prednisone or the equivalent, and NSAIDs with a stable dose. In all trials, the patients were randomized into a placebo group or one of two secukinumab groups, and the baseline characteristics of these groups were comparable.

### 3.3. Results of the Quality Assessment

**Figure 2:**
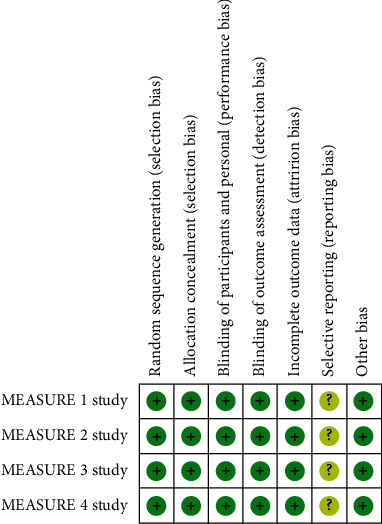
Risk of bias graph: review authors' judgement about each risk of bias item for each included study.

The risk of bias assessment results for the included trials was shown in Figure 2. All trials performed blinding of the AS patients and research investigators. Randomization and concealment of allocation were conducted with effective approaches. There was a low risk of incomplete outcome data and other bias, while the risk of reporting bias was unclear in the 4 RCTs. The quality assessment results for the outcomes were summarized in Table 2.

## 4. Results of the Meta-Analysis

### 4.1. Secukinumab 150 mg versus a Placebo

**Figure 3:**
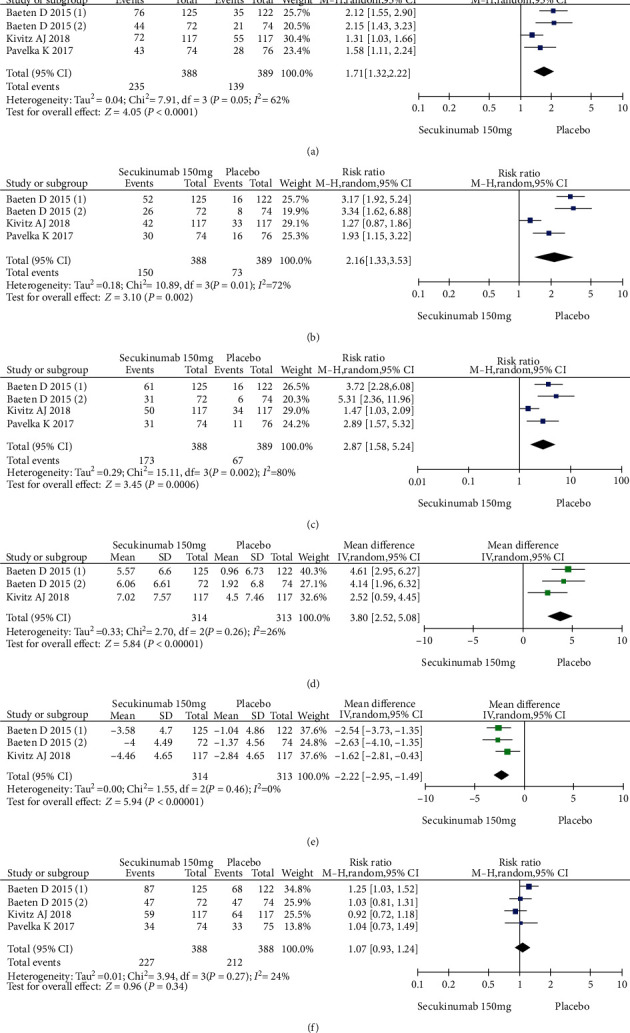
Meta-analysis of secukinumab 150 mg versus placebo at week 16. (a) ASAS 20 response, (b) ASAS 40 response, (c) ASAS 5/6 response, (d) SF-36 PCS score (change from baseline), (e) ASQoL score (change from baseline), (f) AEs. Baeten 2015 (1): MEASURE 1 study, Baeten 2015 (2): MEASURE 2 study.

Four RCTs with 777 patients in the secukinumab 150 mg arm and placebo arm reported the ASAS20, ASAS40, and ASAS5/6 response rates at week 16, and the estimated RRs were 1.71 (95% CI = 1.32 to 2.22, *P* < 0.0001), 2.16 (95% CI = 1.33 to 3.53, *P* = 0.002), and 2.87 (95% CI = 1.58 to 5.24, *P* = 0.0006), respectively (Figure 3). Three trials involving 627 patients published the scores of the SF-36 PCS and ASQoL (mean change from baseline) in the secukinumab 150 mg group and the placebo group, and the estimated MDs were 3.80 (95% CI = 2.52 to 5.08, *P* < 0.00001) and -2.22 (95% CI = −2.95 to -1.49, *P* < 0.00001), respectively (Figure 3). There were significant differences in the above indicators between the two groups. The number of AEs at 16 weeks was considered the safety outcome in our meta-analysis. The results of 776 patients from four RCTs were pooled. The estimated RR was 1.07 (95% CI = 0.93 to 1.24, *P* = 0.34) (Figure 3), showing no significant difference between the two groups. It should be noted that the two experimental arms of NCT02159053 were both secukinumab 150 mg. Therefore, the results from one group (secukinumab 150 mg with no load) with a lower level of heterogeneity were included in our meta-analysis.

### 4.2. Secukinumab 75 mg versus a Placebo

**Figure 4:**
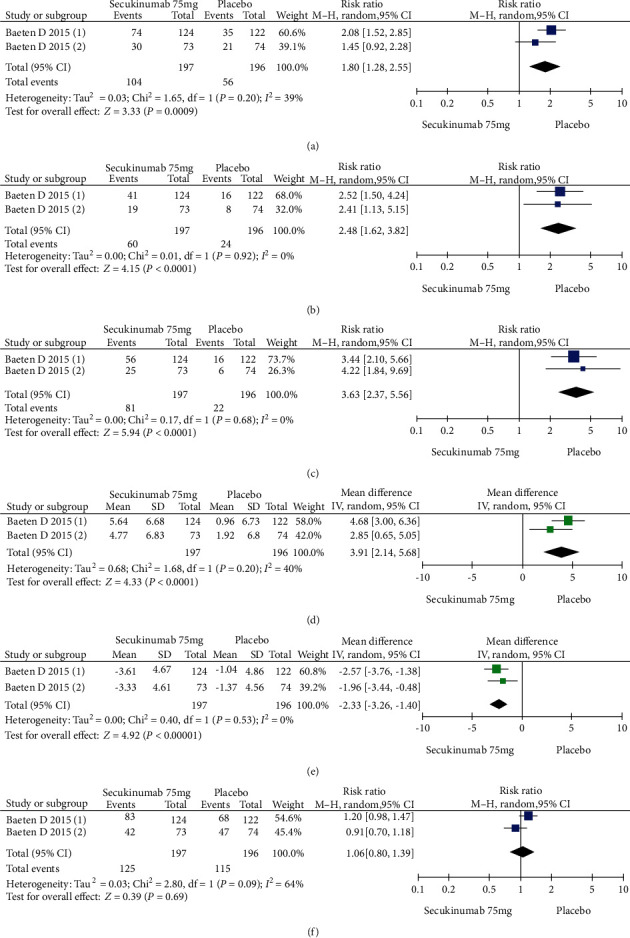
Meta-analysis of secukinumab 75 mg versus placebo at week 16. (a) ASAS 20 response, (b) ASAS 40 response, (c) ASAS 5/6 response, (d) SF-36 PCS score (change from baseline), (e) ASQoL score (change from baseline), (f) AEs. Baeten 2015 (1): MEASURE 1 study, Baeten 2015 (2): MEASURE 2 study.

Two RCTs including 393 patients assessed the efficacy and safety of secukinumab 75 mg and a placebo at week 16. Secukinumab 75 mg showed higher response rates on ASAS20, ASAS40, and ASAS5/6, with estimated RRs of 1.80 (95% CI = 1.28 to 2.55, *P* = 0.0009), 2.48 (95% CI = 1.62 to 3.82, *P* < 0.0001), and 3.63 (95% CI = 2.37 to 5.56, *P* < 0.00001), respectively (Figure 4). For the SF-36 PCS and ASQoL scores (mean change from baseline), the estimated MDs were 3.91 (95% CI = 2.14 to 5.68, *P* < 0.0001) and -2.33 (95% CI = −3.26 to -1.40, *P* < 0.00001), respectively (Figure 4). The meta-analysis revealed significant differences in these indexes between the two groups. In addition, no significant difference was found between the secukinumab 75 mg group and the placebo group in terms of AEs, with an RR of 1.06 (95% CI = 0.80 to 1.39, *P* = 0.69) (Figure 4).

### 4.3. Secukinumab 75 mg versus Secukinumab 150 mg

**Figure 5:**
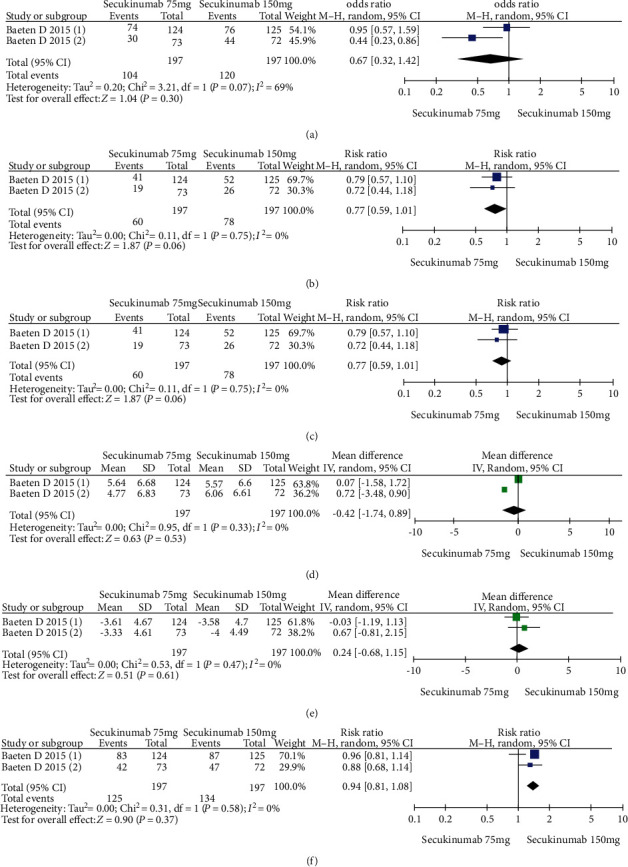
Meta-analysis of secukinumab 75 mg versus 150 mg at week 16. (a) ASAS 20 response, (b) ASAS 40 response, (c) ASAS 5/6 response, (d) SF-36 PCS score (change from baseline), (e) ASQoL score (change from baseline), (f) AEs. Baeten 2015 (1): MEASURE 1 study, Baeten 2015 (2): MEASURE 2 study.

The data from 394 patients from two trials were pooled to compare the efficacy and safety of secukinumab 75 mg versus 150 mg for AS. The estimated RRs of the ASAS20, ASAS40, and ASAS5/6 were 0.67 (95% CI = 0.32 to 1.42, *P* = 0.30), 0.77 (95% CI = 0.59 to 1.01, *P* = 0.06), and 0.89 (95% CI = 0.71 to 1.11, *P* = 0.29), respectively (Figure 5). The estimated MDs of the SF-36 PCS and ASQoL scores (mean change from baseline) were -0.42 (95% CI = −1.74 to 0.89, *P* = 0.53) and 0.24 (95% CI = −0.68 to 1.15, *P* = 0.61), respectively (Figure 5). The pooled results revealed no significant difference in these outcomes between the two groups. In terms of AEs, the estimated RR was 0.94 (95% CI = 0.81 to 1.08, *P* = 0.37) (Figure 5), showing no significant difference between the two groups.

### 4.4. Subgroup Analysis

**Figure 6:**
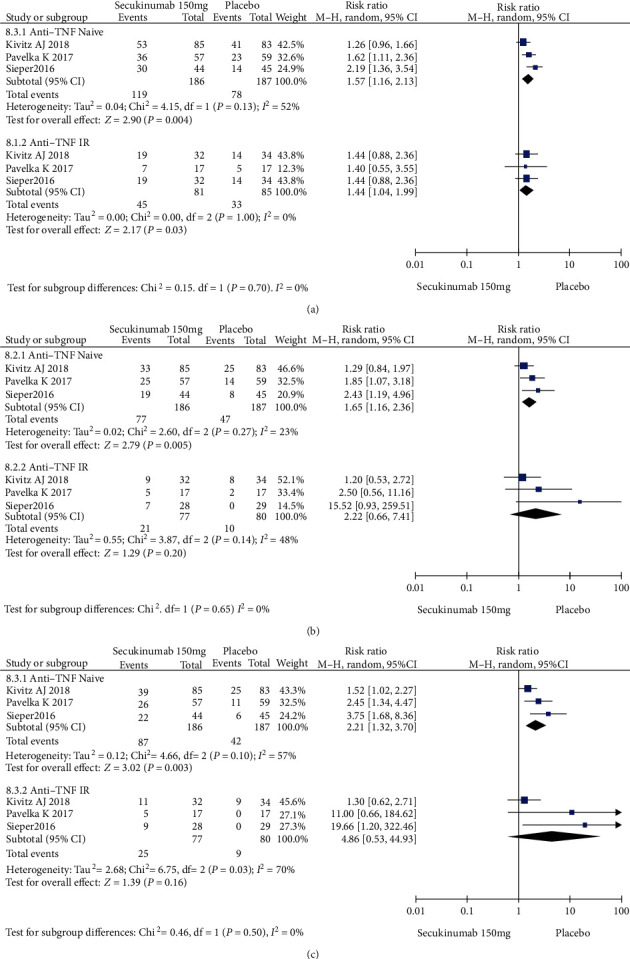
Subgroup analysis of secukinumab 150 mg versus placebo at week 16. (a) ASAS 20 response, (b) ASAS 40 response, (c) ASAS 5/6 response.

To comprehensively evaluate the efficacy of secukinumab 150 mg, subgroup analysis was conducted based on whether the patients previously underwent TNFi treatment. Among the TNFi-naive group, the RRs of the ASAS20, ASAS40, and ASAS5/6 were 1.57 (95% CI = 1.16 to 2.13, *P* = 0.004), 1.65 (95% CI = 1.16 to 2.36, *P* = 0.005), and 2.21 (95% CI = 1.32 to 3.70, *P* = 0.003), respectively, and the differences were statistically significant (Figure 6). In the TNF-IR group, the RRs of the ASAS20, ASAS40, and ASAS5/6 were 1.44 (95% CI = 1.04 to 1.99, *P* = 0.03), 2.22 (95% CI = 0.66 to 7.41, *P* = 0.20), and 4.86 (95% CI = 0.53 to 44.93, *P* = 0.16), respectively, and the magnitudes of improvements in the ASAS40 and ASAS5/6 were statistically nonsignificant (Figure 6).

### 4.5. Sensitivity Analysis

In the meta-analysis of secukinumab 150 mg versus a placebo, the heterogeneity of all outcomes ranged from 0 to 80%. We conducted a sensitivity analysis by removing one single trial each time, and the results suggested that the heterogeneity was mainly attributed to a single study, the study by Kivitz et al. [21].

## 5. Discussion

To our knowledge, this was the first meta-analysis that assessed the short-term efficacy and safety of secukinumab in the management of AS. The present study consisted of three comparisons. In general, improvements in the clinical response and patient-reported outcomes were observed with the secukinumab 150 mg and 75 mg regimens at week 16, and there was no increased risk of AEs. Moreover, the superiority of secukinumab 150 mg over secukinumab 75 mg seemed to be limited, since no significant difference was detected in any endpoint between the two groups.

Four phase 3 studies (MEASURE 1-4) were included in the meta-analysis of secukinumab 150 mg versus a placebo [19–22]. The results showed that the secukinumab 150 mg yielded greater improvements in the clinical response and patient-reported outcomes for AS patients than did a placebo at week 16. The results of most included trials were consistent with those of our meta-analysis, except those of a single study (MEASURE 4) [21], which contributed to significant heterogeneity. After 16 weeks of treatment, in the MEASURE 4 study, the superiority of secukinumab 150 mg over a placebo was inconspicuous, because the patients in the placebo group exhibited greater improvements in efficacy outcomes than did those in other studies. The possible reason for such a higher-than-expected response rate was that under the influence of previous studies, both the patients and investigators might have become increasingly aware of the established efficacy of secukinumab and tended to report better outcomes [21]. In addition, we conducted a subgroup analysis based on whether AS patients previously underwent TNFi treatment. At week 16, secukinumab 150 mg was associated with a significantly higher ASAS20 response rate, regardless of whether the AS patients previously underwent TNFi treatment. In the TNF-IR group, there was a trend that secukinumab 150 mg provided higher ASAS40 and ASAS5/6 response rates, although the magnitudes of the improvements were statistically nonsignificant. These results suggested that secukinumab 150 mg provided benefits for TNFi-naive and TNF-IR patients.

The efficacy of the secukinumab 75 mg regimen for the management of AS was controversial. The results from the MEASURE 1 study showed that secukinumab 75 mg was associated with notable improvements in the clinical response and patient-reported outcomes at week 16 [19]. However, this superiority of the secukinumab 75 mg regimen over a placebo was not observed in the MEASURE 2 study. Because the improvements in all the efficacy outcomes were statistically nonsignificant [19]. These conflicting results might originate from the different methods of administration of secukinumab between the two studies. Obviously, the secukinumab 75 mg group in the MEASURE 1 study received a higher loading dose based on their body weight in the first 4 weeks [19]. This interpretation was supported by our meta-analysis results of secukinumab 75 mg versus 150 mg, which revealed a trend of better efficacy with a higher dose, although no significant difference was detected between the two groups. In addition, secukinumab 300 mg was shown to be more effective than 150 mg for PsA patients [28], suggesting that there might be a dose-dependent effect.

Inflammation and excessive bone formation are two notable characteristics of AS. Although the precise molecular mechanism remains to be fully illustrated, cumulative evidence suggests that IL-17 plays a pivotal role in these two processes. IL-17 is a proinflammatory cytokine that can activate the nuclear factor *κ*B (NF-*κ*B) pathway and enhance the production of various cytokines such as IL-1B, IL-6, IL-8, and TNF-*α* [10]. The release of these cytokines initiates and amplifies the cytokine cascade, leading to uncontrolled inflammation. In addition, IL-17 also contributes to the regulation of bone homeostasis. It has been shown that IL-17 not only promotes osteoclastic differentiation and joint destruction by upregulating the receptor NF-*κ*B ligand (RANKL) but also facilitates osteoblastogenesis and excessive bone formation [29, 30]. These processes occur sequentially rather than in parallel, leading to the progression of AS [4]. Consistent with these results, our meta-analysis further confirms that the IL-23/IL-17 axis is involved in the pathogenesis of AS and that IL-17 is an effective target for the management of AS.

It should be noted that our study had some limitations. First, the sample size of this meta-analysis was relatively small since only four RCTs were included for quantitative synthesis. More large-sample and high-quality RCTs are needed to complete our study. Second, the methods of administration of secukinumab differed across the four trials in the first 8 weeks, which might have influenced the outcomes in the short-term setting. Moreover, the placebo recipients of all trials were switched to the secukinumab group after week 16. To generate meaningful results, our study only focused on the placebo-controlled period. Thus, we were not able to evaluate the long-term efficacy and safety of secukinumab for the management of AS. Finally, our assessment of secukinumab was not comprehensive due to the limited amount of data available. Other outcomes, especially radiographic responses that reflect the progression of AS, should be assessed in future studies.

## 6. Conclusions

Secukinumab is effective in treating AS and generally well tolerated by AS patients in the short term, regardless of whether the patients previously underwent TNFi treatment. The superiority of secukinumab 150 mg over secukinumab 75 mg seems to be limited. However, only four trials were included in the statistical analysis, and more large-sample and high-quality RCTs are needed in the future to further evaluate the efficacy and safety of secukinumab for the management of AS.

## Figures and Tables

**Table 1:** Basic characteristics of all eligible studies.

ClinicalTrials.gov numbers	Study (y)	Study design	Treatment	Samples	Male *N* (%)	Weight (kg)	Age (years)	Duration of AS (years)	Outcomes
NCT01358175	Baeten (2015) [19]	Randomized double-blind placebo-controlled multicenter phase 3 trials	SEC 75 mg SEC 150 mg Placebo MEASURE 1	124 125 122	88 (71) 84 (67) 85 (70)	77.7 ± 19.6 74.7 ± 16.2 76.7 ± 14.4	42.3 ± 13.2 40.1 ± 11.6 43.1 ± 12.4	7.9 ± 9.7 6.5 ± 6.9 8.3 ± 8.9	a b c d e f at week 16
NCT01649375	Baeten (2015) [19] Sieper (2016) [22]	Randomized double-blind placebo-controlled multicenter phase 3 trials	SEC 75 mg SEC 150 mg Placebo MEASURE 2	73 72 74	51 (70) 46 (64) 56 (76)	81.5 ± 17.4 82.3 ± 18.0 80.3 ± 15.2	44.4 ± 13.1 41.9 ± 12.5 43.6 ± 13.2	5.3 ± 7.4 7.0 ± 8.2 6.4 ± 8.9	a b c d e f at week 16
NCT02008916	Pavelka (2017) [20]	Randomized double-blind placebo-controlled multicenter phase 3 trials	SEC 150 mg SEC 300 mg Placebo MEASURE 3	74 76 76	46 (62) 50 (66) 40 (53)	80.3 ± 19.2 82.7 ± 16.9 79.0 ± 15.5	42.9 ± 11.1 42.1 ± 11.8 42.7 ± 11.4	6.0 ± 7.2 5.3 ± 7.3 5.2 ± 6.4	a b c f at week 16
NCT02159053	Kivitz (2018) [21]	Randomized double-blind placebo-controlled multicenter phase 3 trials	SEC 150 mg no load SEC 150 mg load Placebo MEASURE 4	117 116 117	83 (71) 81 (70) *n* (65)	80.3 ± 18.2 83.4 ± 20.4 80.6 ± 17.1	41.2 ± 11.1 44.5 ± 11.6 43.4 ± 12.5	6.5 ± 7.6 8.4 ± 10.8 7.1 ± 9.2	a b c d e f at week 16

Measure 1: intravenous secukinumab (10 mg/kg) or matched placebo at weeks 0, 2, and 4, followed by subcutaneous secukinumab (150 mg or 75 mg) or matched placebo every 4 weeks starting at week 8. Measure 2: subcutaneous secukinumab (150 mg or 75 mg) or matched placebo at baseline; at weeks 1, 2, and 3; and every 4 weeks starting at week 4. Measure 3: intravenous secukinumab (10 mg/kg) or matched placebo at weeks 0, 2, and 4, followed by subcutaneous secukinumab (300 mg or 150 mg) or matched placebo every 4 weeks starting at week 4. Measure 4:subcutaneous secukinumab (150 mg) with loading dose (150 mg), without loading dose or placebo at weeks 1, 2, and 3 and every 4 weeks starting at week 4. (a) ASAS20 response, (b) ASAS 40 response, (c) ASAS5/6 response, (d) SF-36 PCS score (change from baseline), (e) ASQoL score (change from baseline).

**Table 2:** The GRADE evidence quality for each outcome.

Outcomes	No. of included trials	No. of patients		MD or RR (95% CI)	Heterogeneity	GRADE
SEC150mg VS PLB		SEC150mg	PLB			
ASAS20 response	4	388	389	1.71 (1.32, 2.22)	*I* ^2^ = 62%, *P* = 0.05	Moderate
ASAS40 response	4	388	389	2.16 (1.33, 3.53)	*I* ^2^ = 72%, *P* = 0.01	Moderate
ASAS5/6 response	4	388	389	2.87 (1.58, 5.24)	*I* ^2^ = 80%, *P* = 0.002	Low
SF-36 PCS	3	314	313	3.80 (2.52,5.08)	*I* ^2^ = 26%, *P* = 0.26	High
ASQOL	3	314	313	−2.22 (-2.95, −1.49)	*I* ^2^ = 0%, *P* = 0.46	High
AE	4	388	388	1.07 (0.93, 1.24)	*I* ^2^ = 24%, *P* = 0.27	High
SEC75mg VS PLB		SEC75mg	PLB			
ASAS20 response	2	197	196	1.80 (1.28, 2.55)	*I* ^2^ = 39%, *P* = 0.20	Moderate
ASAS40 response	2	197	196	2.48 (1.62, 3.82)	*I* ^2^ = 0%, *P* = 0.92	Moderate
ASAS5/6 response	2	197	196	3.63 (2.37, 5.56)	*I* ^2^ = 0%, *P* = 0.68	Low
SF-36 PCS	2	197	196	3.91 (2.14, 5.68)	*I* ^2^ = 40%, *P* = 0.20	Moderate
ASQOL	2	197	196	−2.33 (−3.26, −1.40)	*I* ^2^ = 0%, *P* = 0.53	Moderate
AE	2	197	196	1.06 (0.80, 1.39)	*I* ^2^ = 64%, *P* = 0.09	Low
SEC75mg VS SEC150mg		SEC75mg	SEC150mg			
ASAS20 response	2	197	197	0.67 (0.32, 1.42)	*I* ^2^ = 69%, *P* = 0.07	Low
ASAS40 response	2	197	197	0.77 (0.59, 1.01)	*I* ^2^ = 0%, *P* = 0.75	Moderate
ASAS5/6 response	2	197	197	0.89 (0.71, 1.11)	*I* ^2^ = 0%, *P* = 0.54	Moderate
SF-36 PCS	2	197	197	−0.42 (−1.74, 0.89)	*I* ^2^ = 0%, *P* = 0.33	Low
ASQOL	2	197	197	0.24 (−0.68, 1.15)	*I* ^2^ = 0%, *P* = 0.47	Moderate
AE	2	197	197	0.94 (0.81, 1.08)	*I* ^2^ = 0%, *P* = 0.58	Moderate

## Data Availability

All data generated and analyzed during the study are available from the corresponding author upon request.
